# Metanephric adenoma in a pediatric patient case report

**DOI:** 10.3389/fped.2025.1539220

**Published:** 2025-04-03

**Authors:** Şule Çalışkan Kamış, Begül Yağcı, Ayşe Selcan Koç, Zeynel Abidin Taş

**Affiliations:** ^1^ Department of Pediatric Hematology and Oncology, University of Health Sciences, Adana Faculty of Medicine, Adana City Education and Research Hospital, Adana, Türkiye; ^2^ Department of Radiology, University of Health Sciences, Adana Faculty of Medicine, Adana City Education and Research Hospital, Adana, Türkiye; ^3^ Department of Medical Pathology, University of Health Sciences, Adana Faculty of Medicine, Adana City Education and Research Hospital, Adana, Türkiye

**Keywords:** metanephric adenoma, Wilms tumor, BRAF v600E mutation, case report metanephric adenoma, BRAF v600E mutation 38

## Abstract

Metanephric adenoma (MA) is a rare benign renal tumor, with an incidence of 0.2%–1%. Approximately 90% of MA cases present with the BRAF V600E mutation. This study reports an 8-year-old male child who presented with abdominal pain for one month. Abdominal ultrasound revealed a cystic necrotic mass measuring 56 × 45 mm in the right kidney. A preliminary diagnosis of Wilms tumor (WT) led to the initiation of preoperative vincristine therapy. Right nephroureterectomy was performed by pediatric surgery. Histopathological analysis could not differentiate between MA and WT. Immunohistochemical findings were positive for WT1, PANCK (weak focal), INI1 (intact), PAX8, CD56, and CD57. Genetic testing confirmed the presence of the BRAF V600E mutation (1799T > A, 1799_1800TG > AA). The patient was diagnosed with MA and was followed without chemotherapy. In conclusion, MA, which can be mistaken for WT, should be considered in the differential diagnosis of pediatric renal neoplasms. Immunohistochemical evaluation and genetic testing are essential for a definitive diagnosis.

## Introduction

Metanephric adenoma (MA) is a rare benign renal tumor, often misdiagnosed as Wilms tumor (WT), particularly in pediatric cases. Despite being exceedingly rare in children, there is a limited number of case reports in the literature (1, 2). The incidence of MA accounts for approximately 0.2%–1% of all renal tumors (3). While MA is predominantly observed in adults, several pediatric cases have also been documented (4). In most instances, the tumor is asymptomatic and is typically discovered incidentally during radiological imaging (5). Epidemiological studies indicate that MA occurs more frequently in females than males (6). Around 90% of MA cases have been linked to the BRAF V600E mutation, providing further insight into the molecular mechanisms underlying the tumor (7). The treatment of choice for MA is nephron-sparing surgery, which aims to preserve renal function and minimize the loss of healthy kidney tissue (8).

## Case report

**Figure 1:**
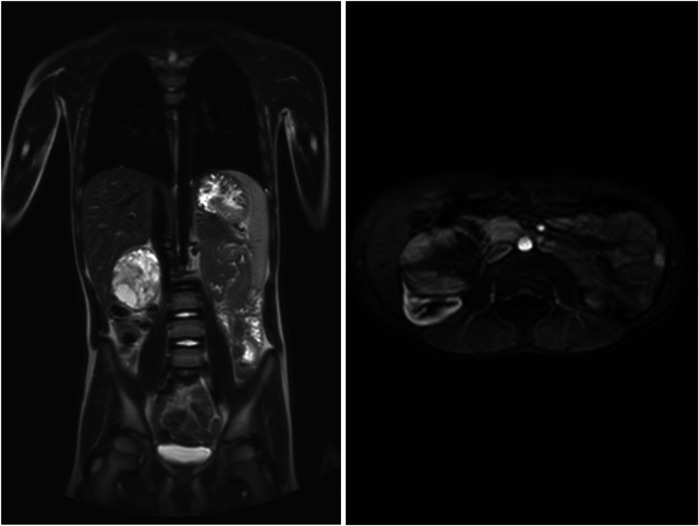
Metanephric adenoma in magnetic resonance image.

**Figure 2:**
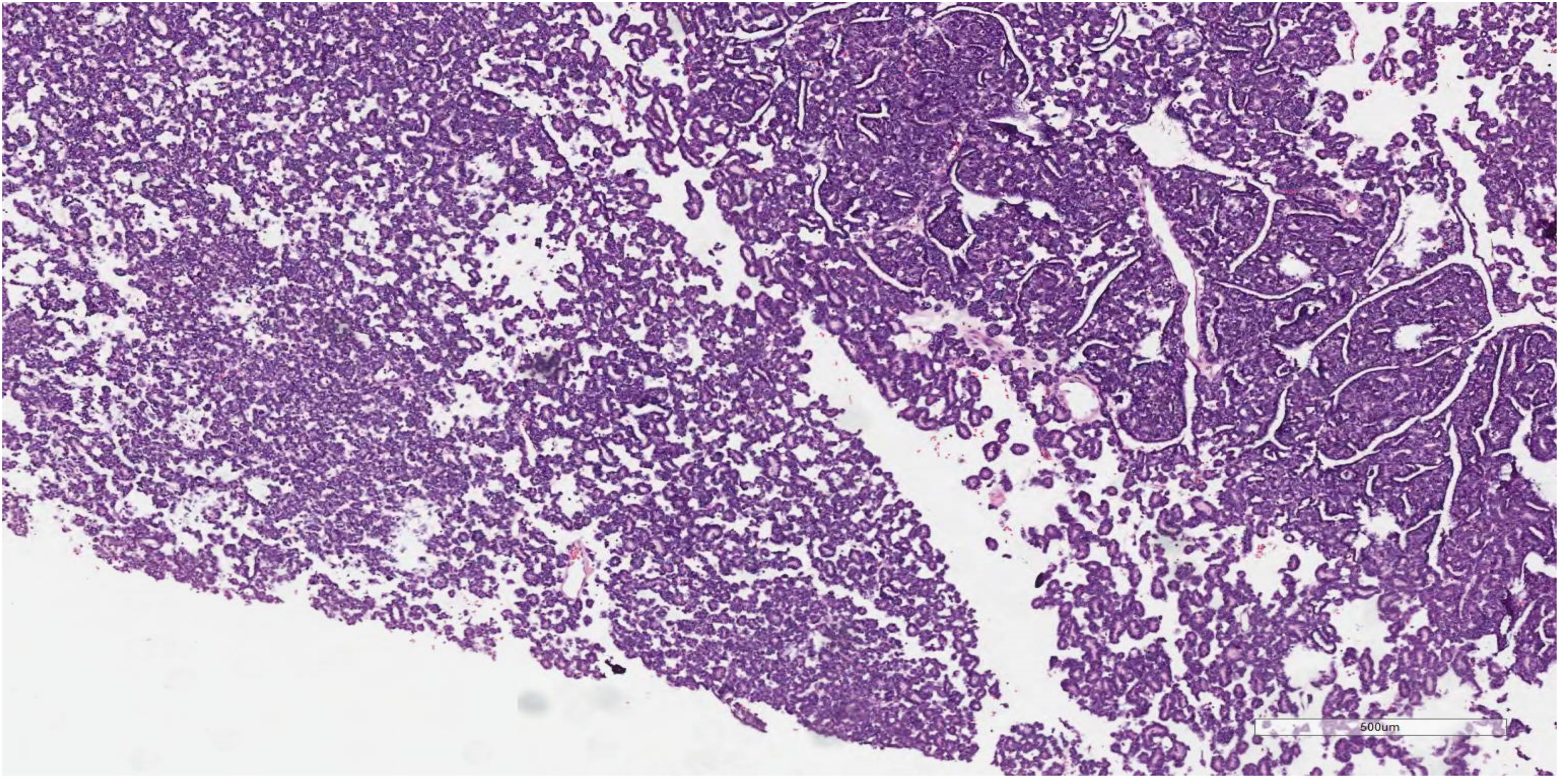
Hematoxylin-eosin (H&E) stained pathology section of metanephric adenoma. The section reveals clusters of atypical epithelial cells forming papillary or glandular structures (on the right side of the field). These cells exhibit features such as enlarged, hyperchromatic nuclei and occasional nuclear overlap, suggesting malignant transformation. The surrounding areas (on the left side) contain more loosely arranged cells and possible necrotic or hemorrhagic debris, indicating invasive growth into the adjacent tissue (Scale bar = 100 µm).

**Figure 3:**
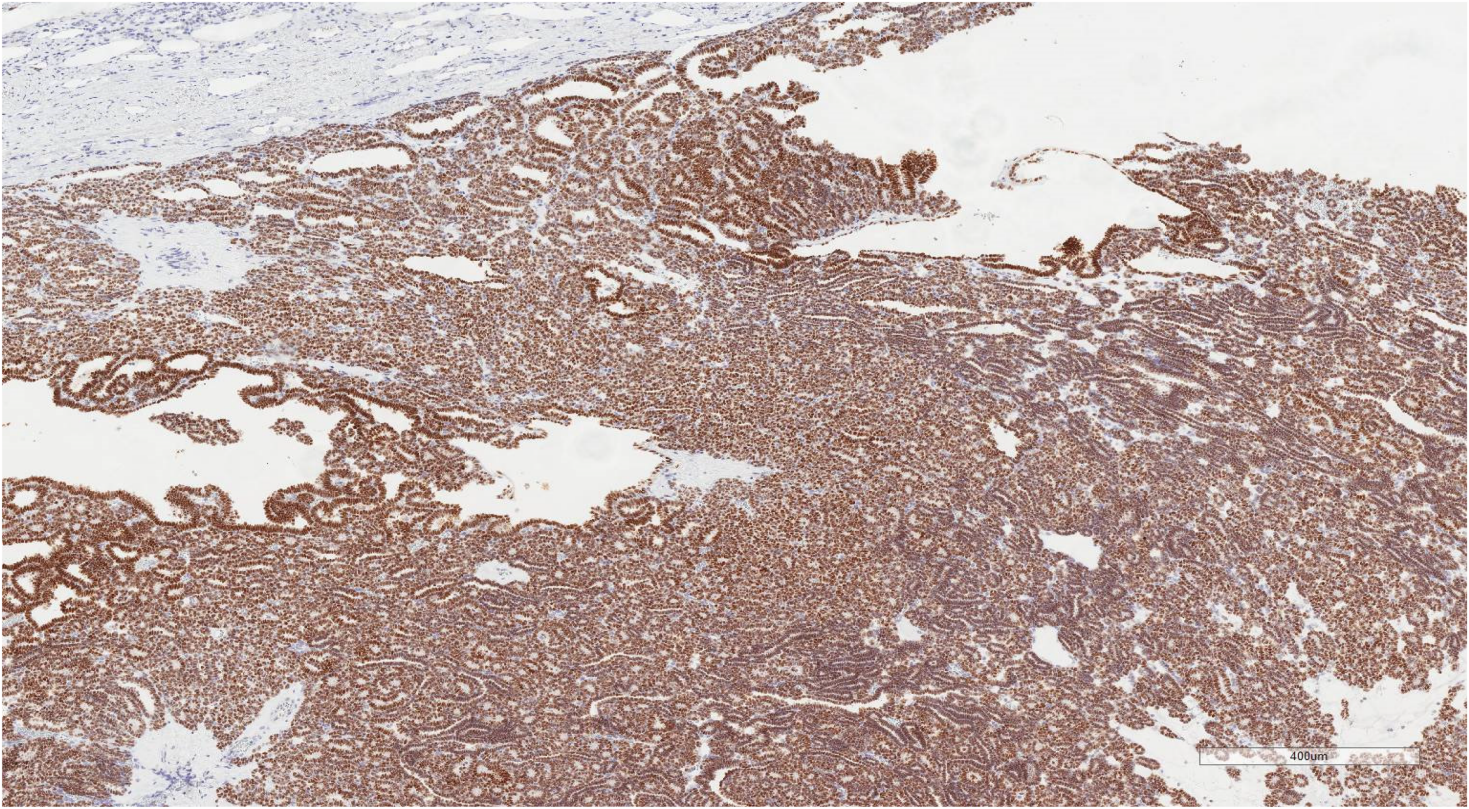
Wt1 immunohistochemical staining of metanephric adenoma. Diffuse and strong nuclear WT1 positivity is observed throughout the tumor tissue, confirming the diagnosis of metanephric adenoma. The staining pattern highlights the characteristic histological architecture of the tumor (Scale bar = 400 µm).

**Figure 4:**
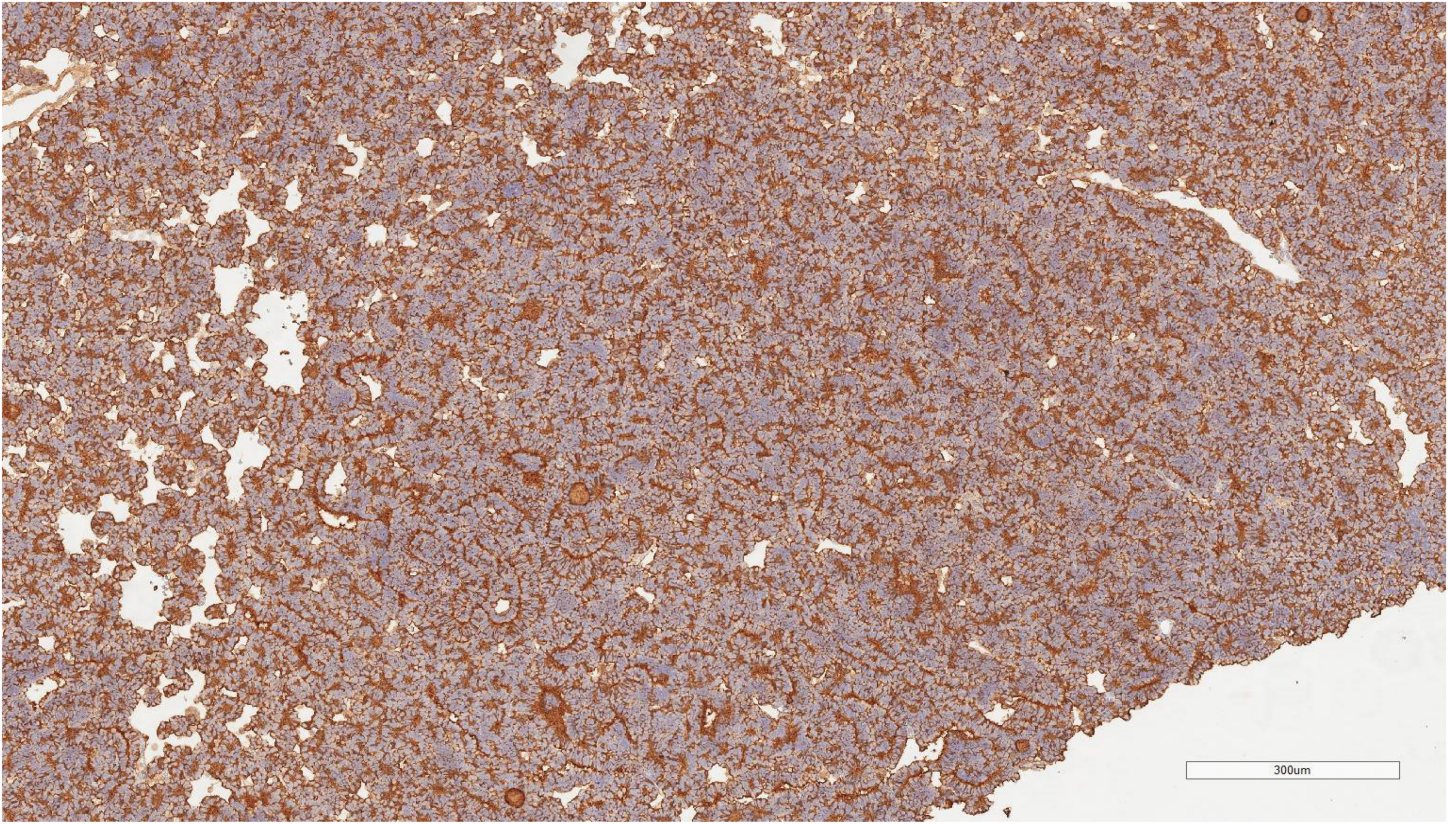
Cd57 immunohistochemical staining of metanephric adenoma. The tumor exhibits diffuse and strong membranous and cytoplasmic CD57 positivity, supporting the diagnosis of metanephric adenoma. The staining pattern highlights the characteristic cellular distribution within the tumor tissue (Scale bar = 300 µm).

An 8-year-old boy presented with a 1-month history of abdominal pain. Tenderness was detected in the abdomen during physical examination. His medical history did not reveal any specific findings. Abdominal ultrasonography (USG) showed a solid lesion in the lower pole of the right kidney, measuring 56 × 45 mm, with cystic necrotic areas. Contrast-enhanced abdominal magnetic resonance imaging (MRI) revealed a 56 × 51 mm exophytic mass located in the lower pole of the right kidney, exhibiting heterogeneous signal intensity, heterogeneous enhancement, and occasional diffusion restriction (Figure 1). The patient was started on preoperative vincristine treatment. A right nephroureterectomy was performed by the pediatric surgery department. Histopathological examination could not differentiate between metanephric adenoma (MA) and Wilms tumor. Immunohistochemical staining revealed WT1 (+), PANCK weak focal (+), INI1 intact, PAX8 (+), CD56 (+), CD57 (+), and synaptophysin (−) staining (Figures 2–4). The Ki67 proliferation index was 10%–12%. The pathology blocks were sent to a reference center for confirmation, where the upper central pathology was evaluated as MA. Immunohistochemical examination showed diffuse strong membranous staining with the CD57 antibody. To further confirm the diagnosis of MA, genetic testing was performed to detect the BRAF V600E mutation. Genetic results revealed the presence of the V600E (1799T > A) and V600E complex (1799_1800TG > AA) mutations. The patient was diagnosed with MA, and a plan was made for follow-up without chemotherapy.

### Follow-up

The patient was diagnosed on July 18, 2023, and has been under regular follow-up for the past 22 months. The most recent abdominal ultrasonography, performed on February 19, 2025, confirmed the absence of the right kidney postoperatively. The left kidney measured 85 mm in the longitudinal axis, with a parenchymal thickness of 11 mm and normal echogenicity. No signs of dilation were observed in the collecting system. Laboratory investigations revealed a lactate dehydrogenase (LDH) level of 207 IU, which is within the normal reference range (110–295 IU). Urinalysis showed no erythrocytes or leukocytes. To date, there has been no evidence of metastasis or recurrence.

Although metanephric adenoma is typically associated with an excellent prognosis, isolated cases of metastatic progression have been reported in the literatüre (9, 10). Therefore, ongoing surveillance remains essential. Our patient continues to be monitored at regular intervals, with no adverse clinical findings observed to date.

## Discussion

Metanephric adenoma (MA) is a rare benign renal tumor with a typically slow clinical progression. While it is often asymptomatic, non-specific symptoms, such as abdominal pain, may occasionally be observed (9). MA constitutes approximately 0.2% of renal epithelial malignancies (11). Notably, the incidence of MA is higher in females compared to males (12). Although MA is most commonly diagnosed in adults, pediatric cases remain exceedingly rare (13). The radiological appearance of MA often mimics that of Wilms tumor (WT), which can complicate diagnosis (9). The presence of the BRAF V600E mutation is a distinguishing feature in about 90% of MA cases (14). Immunohistochemically, MA typically shows positivity for WT1 and CD57 (15), which is consistent with the findings in our case.

In a case series by Netto et al. (2007), a 2-year-old girl was diagnosed with MA, further emphasizing its rare presentation in the pediatric population (16). Similarly, de Jel et al. (17) detected the BRAF V600E mutation in three out of 41 MA cases, highlighting the importance of genetic testing in the diagnosis of this rare tumor. Furthermore, Mei et al. (18) followed a 2-year-old child with MA for 14 months and reported no recurrence or metastasis, reinforcing the generally indolent nature of this tumor in pediatric patients. Our case also demonstrated positivity for WT1 and CD57, with the BRAF V600E mutation confirmed genetically, further supporting the molecular characteristics of MA.

The genetic confirmation of MA, especially the detection of the BRAF V600E mutation, is essential for accurate diagnosis and management, particularly given its potential to be confused with more common renal tumors like Wilms tumor (19). Genetic and immunohistochemical evaluation are indispensable tools in diagnosing rare renal neoplasms such as MA, ensuring that appropriate treatment strategies are employed.

## Conclusion

In the differential diagnosis of renal neoplasms in children, it is critical to consider MA, which may be mistaken for Wilms tumor due to their similar presentation. While MA remains exceedingly rare in the pediatric population, the importance of immunohistochemical evaluation and genetic testing for definitive diagnosis cannot be overstated. These diagnostic approaches ensure that MA is accurately identified and differentiated from other renal tumors, facilitating appropriate clinical management.

## Data Availability

The data is available upon reasonable request.
